# Bioactivity-Guided Isolation of Secondary Metabolites with Antioxidant and Antimicrobial Activities from *Camellia fascicularis*

**DOI:** 10.3390/foods13142266

**Published:** 2024-07-18

**Authors:** Ruonan Li, Jiandong Tang, Jingjing Li, Boxiao Wu, Junrong Tang, Huan Kan, Ping Zhao, Yingjun Zhang, Weihua Wang, Yun Liu

**Affiliations:** 1 Key Laboratory of Forest Resources Conservation and Utilization in the Southwest Mountains of China Ministry of Education, Southwest Forestry University, Kunming 650224, China; lrn@swfu.edu.cn (R.L.); hdyknctjd23@swfu.edu.cn (J.T.); kiki0908222@swfu.edu.cn (J.L.); wbx1437@swfu.edu.cn (B.W.); tjrzy2016@swfu.edu.cn (J.T.); kanhuan@swfu.edu.cn (H.K.); hypzhao@swfu.edu.cn (P.Z.); 34016@ztu.edu.cn (W.W.); 2 State Key Laboratory of Phytochemistry and Plant Resources in West China, Kunming Institute of Botany, Chinese Academy of Sciences, Kunming 650224, China; zhangyj@mail.kib.ac.cn

**Keywords:** *Camellia fascicularis*, bioactivity-guided isolation, secondary metabolites, antioxidant, antimicrobial

## Abstract

*Camellia fascicularis* has important ornamental, medicinal, and food values, which also have tremendous potential for exploiting bioactivities. We performed the bioactivity-guided (antioxidant and antimicrobial) screening of eight fractions obtained from the ethyl acetate phase of *C. fascicularis*. The antioxidant activity was measured by DPPH, ABTS, and FRAP, and the antibacterial activity was measured by the minimum inhibitory concentration (MIC) of *Pseudomonas aeruginosa*, *Escherichia coli,* and *Staphylococcus aureus*. The results of bioactivity-guided isolation indicated that the major antioxidant compounds in the ethanolic extracts of *C. fascicularis* may be present in fractions (Fr.) (A–G, obtained after silica gel column chromatography). Fr. (D–I, obtained after silica gel column chromatography) is a fraction of *C. fascicularis* with antimicrobial activity. The structures of compounds were determined by spectral analysis and nuclear magnetic resonance (NMR) combined with the available literature on secondary metabolites of *C. fascicularis* leaves. In this study, 17 compounds were identified, including four phenolics (**1**, **3**–**4**, and **14**), a phenylpropane (**2**), five terpenoids (**5**–**7**, **12**, and **15**), four flavonoids and flavonoid glycosides (**8**–**10** and **16**), and two lignins (**13** and **17**). Compounds **4**–**7**, **13**–**15**, and **17** were isolated from the genus *Camellia* for first time. The remaining compounds were also isolated from *C. fascicularis* for first time. The evaluation of antioxidant and antimicrobial activities revealed that compounds **1**, **3**, **9**, **11,** and **17** exhibited higher antioxidant activity than the positive control drug (ascorbic acid), and compounds **4**, **8, 10**, and **13** showed similar activity to ascorbic acid. The other compounds had weaker or no significant antioxidant activities. The MIC of antibacterial activity for compounds **4**, **7**, and **11**–**13** against *P*. *aeruginosa* was comparable to that of the positive control drug tetracycline at 125 µg/mL, and other secondary metabolites inhibited *E*. *coli* and *S*. *aureus* at 250–500 µg/mL. This is also the first report of antioxidant and antimicrobial activities of compounds **5**–**7**, **13**–**15**, and **17**. The results of the study enriched the variety of secondary metabolites of *C. fascicularis* and laid the foundation for further research on the pharmacological efficacy and biological activity of this plant.

## 1. Introduction

*Camellia fascicularis*, a genus of *Camellia* in the family Theaceae, is an endemic plant in Yunnan province, China. *C. fascicularis*, which is a rare species resource with unique golden petals and is also known as “giant panda in the plant kingdom”, “queen of the tea family”, and “living fossil of plants”, was first discovered in the Hekou County and was only distributed in the Gejiu, Maguan, and Hekou counties [1]*. C. fascicularis* leaves possess high amino acid and mineral content and are considered as an edible plant resource with high nutritional and health values [2,3]. There is limited literature on the metabolites of *Camellia*, with polyphenols (catechin), flavonoids (flavonoids are classified into various types depending on their chemical structure, degree of unsaturation, and oxidation of carbon ring, including flavones, flavanones, isoflavones, flavonols, chalcones, flavanols, and anthocyanins; each of these flavonoids is widely distributed in nature), saponins (mainly flavonoids combined with glycosides to form flavonoid glycosides and oleanoid-type pentacyclic triterpenoid saponins), and steroids (mainly in the form of steroids and steroidal saponins), which are the involved primary active components [4,5,6,7,8]. It has been proved by experiments that the flowers and leaves of *C. fascicularis,* in addition to being used as tea, have many pharmacological effects such as antioxidant [9], anti-tumour [10,11], and anti-inflammatory [12,13]. Due to its limited distribution in Yunnan, the investigation into secondary metabolites of *C. fascicularis* commenced relatively late; however, it holds immense potential for exploring the biological activities of *C. fascicularis*.

The isolation and identification of monomeric active compounds from complex plant components are a pivotal objective in the field of natural product chemistry research [14]. Activity evaluation-oriented chemical component isolation is an effective method to discover and identify phytochemical active components in the process of chemical component isolation and purification, activity evaluation is used to locate the parts or fractions where the plant active components are located, and targeted isolation and purification is carried out on the identified active parts or streams to ultimately obtain the chemical active components of the Traditional Chinese Medicine [15]. This greatly improves the separation efficiency of the active ingredients, and many structurally defined plant active ingredients are obtained by this method [16]. The significance of the existing research findings is considered in exploring the bioactivities of *C. fascicularis*. Therefore, the present study was oriented towards antioxidant and antibacterial activities, using a combination of multiple spectral separation techniques to isolate and purify the antioxidant and antibacterial active components from *C. fascicularis*, and their structural identification was carried out to preliminarily explore the structure–activity relationships in order to provide a theoretical reference and basis.

## 2. Materials and Methods

### 2.1. Instrumentation

The following instruments were utilized in the experiments: Bruker AV 500 MHz Nuclear Magnetic Resonance Instrument (Bruker, Saarbrucken, Germany), XEVO G2-XS Q-Tof High-Resolution Mass Spectrometer (Waters, Milford, MA, USA), NP7000 Semi-preparative Liquid Phase (Jiangsu Hanbang Technology Co., Ltd., Huaian, China), AX224ZH\E Electronic Balance (Ohaus Instruments, Changzhou Co., Ltd., Changzhou, China), N-1300 Rotary Evaporator, CA-111 Cold Trap (Shanghai Ailang Instrument Co., Ltd., Shanghai, China), SHZ-DIII Circulating Water Vacuum Pump (Gongyi Yu Hua Instrument Co., Ltd., Gongyi, China), SpectraMax 190 enzyme labeller (Molecular Devices Co., Ltd., Shanghai, China), ZQZY-CF9.9 oscillating incubator (Shanghai Zhichu Instrument Co., Ltd., Shanghai, China), and ZF-7 triple-use UV analyser (Shanghai Jiapeng Science and Technology Co., Ltd., Shanghai, China).

### 2.2. Chemicals and Reagents

2,2-Diphenyl-1-picrylhydrazyl (DPPH), 2,2′-azino-bis(3-ethylbenzothiazoline-6-sulfonic acid) (ABTS), 2,4,6-tris(2-pyridyl)-S-triazine, (TPTZ), and ascorbic acid were obtained from Beijing Solarbio Science & Technology Co., Ltd. (Beijing, China). Methanol (HPLC grade) was purchased from Shanghai Xingke High Purity Solvent Co., Ltd. (Shanghai, China). Dimethyl sulfoxide (DMSO) and all other chemicals of analytical grade were purchased from Sinopharm Chemical Reagent Co., Ltd. (Shanghai, China). Macroporous resin D101 and Sephadex LH–20 were purchased from Shanghai Yuanye Biotechnology Co., Ltd. (Shanghai, China), column chromatography silica gel (200–300 mesh and 300–400 mesh) and thin-layer chromatography silica gel plates were purchased from Qingdao Ocean Chemical Co., Ltd. (Qingdao, China), and middle chromatogram isolated (MCI) reversed-phase column was purchased from Beijing Lvbaicao Technology Development Co., Ltd. (Beijing, China). The reagents (industrial grade) used in the column chromatography process were purchased from Yunnan Liyan Technology Co Ltd. (Kunming, China).

### 2.3. Plant Material

The voucher specimen (52860) of *C. fascicularis* was identified by taxonomist Min Tianlu and preserved in the Herbarium of Kunming Institute of Botany, Chinese Academy of Sciences. The leaves of *C. fascicularis* used in this experiment were obtained from Dawei Mountain Nature Reserve, Hekou County, Yunnan Province, China, in December 2019, and were identified as *C. fascicularis* by taxonomist Prof. Xiang Jianying of Southwest Forestry University.

### 2.4. Extraction and Isolation

A total of 10.7 kg of the dried *C. fascicularis* leaf sample was pulverized to about 40 mesh and then extracted three times with 95% methanol at 50 °C for 3, 2, and 1 h each time, respectively. After combining the extracts and evaporating the solvents under low pressure at 50 °C, the methanolic extract of *C. fascicularis* (868.3 g) was obtained. Subsequently, 5 L of distilled water was added for thorough mixing, followed by three extractions using an equal volume of industrial ethyl acetate. The ethyl acetate phase extract (177.8 g) was obtained through low-pressure rotary evaporation at 40 °C. The ethyl acetate phase extract was mixed with (267.0 g) of macroporous resin and loaded onto the column and eluted with a gradient of CH_3_OH:H_2_O (0:1→1:0), and the same portions were combined by thin-layer chromatography (TLC) detection as 4 fractions (Fr. I–IV). After the preliminary screening of Fr. (I–IV) for TLC and antioxidant activity, Fr. (II–III) was found to have good TLC site formation and better antioxidant activity than the other fractions (DPPH IC_50_: 69.44 ± 2.92/µg/mL; ABTS IC_50_: 151.23 ± 12.75/µg/mL). The combined portion of (45.0 g) (Fr. II–III) of the extract was subjected to silica gel column chromatography, with the gradient elution of CHCl_3_:CH_3_OH (1:0→0:1), and the same portion of the combined fractions was obtained by TLC detection as 8 fractions [17] (Table 1).

Fr. A (V_CHCl3:CH3OH_ = 50:1) elution fraction (6.0 g) was separated by MCI reversed-phase column chromatography and gradient elution with MeOH:H_2_O (2:3, 1:1, 3:2, 7:3, 4:1, 9:1, 10:0, *v*/*v*) to obtain 9 flow fractions (Aa–Ai). Fr. Ah was further purified, and impurities were removed using Sephadex LH–20 (CH_2_Cl_2_:MeOH = 95:5), followed by separation by elution with small orthophase silica gel (CH_2_Cl_2_:MeOH), and finally purified using preparative high-performance liquid chromatography (PHPLC) to give monomeric compounds **1** (5.1 mg, V_methanol:water_ = 53:47, *t*_R_ = 15 min), **2** (4.5 mg, V_methanol:water_ = 78:22, *t*_R_ = 7 min), and **3** (7.1 mg, V_methanol:water_ = 59:41, *t*_R_ = 10 min). Fr. Aa was further purified, and impurities were removed using Sephadex LH–20 (CH_2_Cl_2_:MeOH = 95:5), followed by separation by elution with small orthophase silica gel (CH_2_Cl_2_:MeOH), and finally purified using PHPLC to give monomeric compound **5** (3.5 mg, V_methanol:water_ = 23:77, *t*_R_ = 33 min). Fr. Ac was further purified, and impurities were removed using Sephadex LH–20 (CH_2_Cl_2_:MeOH = 95:5), followed by separation by elution with small orthophase silica gel (CH_2_Cl_2_:MeOH), and finally purified using PHPLC to give monomeric compounds **4** (4.2 mg, V_methanol:water_ = 37–87, *t*_R_ = 12 min), **6** (3.9 mg, V_methanol:water_ = 20–100, *t*_R_ = 26 min), and **7** (2.4 mg, V_methanol:water_ = 35–95, *t*_R_ = 20 min).

Fr. F (V_CHCl3:CH3OH_ = 5:1) elution fraction (5.0 g) was separated by MCI reversed-phase column chromatography and gradient elution with MeOH:H_2_O (2:3, 1:1, 3:2, 7:3, 4:1, 9:1, 10:0, *v*/*v*) to obtain 9 flow fractions (Fa–Fi). Fr. Fi was further purified, and impurities were removed using Sephadex LH–20 (MeOH:H_2_O = 95:5) to give compound **10** (4.2 mg), and finally purified using PHPLC to give monomeric compounds **8** (3.5 mg, V_methanol:water_ = 76:24, *t*_R_ = 13 min) and **9** (4.2 mg, V_methanol:water_ = 43:57, *t*_R_ = 8 min). Fr. Fc was further purified, and impurities were removed using Sephadex LH–20 (MeOH:H_2_O = 95:5), and finally purified using PHPLC to give monomeric compound **11** (3.4 mg, V_methanol:water_ = 63:37, *t*_R_ = 14 min). Fr. Fd was further purified, and impurities were removed using Sephadex LH–20 (MeOH:H_2_O = 95:5), and finally purified using PHPLC to give monomeric compounds **12** (4.5 mg) and **13** (10.3 mg, V_methanol:water_ = 37:63, *t*_R_ = 14 min). Fr. Fa was further purified, and impurities were removed using Sephadex LH–20 (MeOH:H_2_O = 95:5), and finally purified using PHPLC to give monomeric compound **14** (9.3 mg, V_methanol:water_ = 27:73, *t*_R_ = 19 min).

Fr. G (V_CHCl3:CH3OH_ = 2:1) elution fraction (9.0 g) was separated by MCI reversed-phase column chromatography and gradient elution with MeOH:H_2_O (2:3, 1:1, 3:2, 7:3, 4:1, 9:1, 10:0, *v*/*v*) to obtain 9 flow fractions (Ga–Gi). Fr. Gh was further purified, and impurities were removed using Sephadex LH–20 (CH_2_Cl_2_:MeOH = 1:1), followed by separation by elution with small orthophase silica gel (CH_2_Cl_2_:MeOH), and finally purified using PHPLC to give monomeric compound **15** (3.4 mg, V_methanol:water_ = 93:7, *t*_R_ = 8 min). Fr. Gd was further purified and decontaminated using Sephadex LH–20 (CH_2_Cl_2_: MeOH = 1:1) to give compound **16** (3.6 mg).

Fr. D (V_CHCl3:CH3OH_ = 15:1) elution fraction (4.0 g) was separated by MCI reversed-phase column chromatography and gradient elution with MeOH:H_2_O (2:3, 1:1, 3:2, 7:3, 4:1, 9:1, 10:0, *v*/*v*) to obtain 8 flow fractions (Da–Dh). Fr. Dc was further purified, and impurities were removed using Sephadex LH–20 (CH_2_Cl_2_:MeOH = 95:5), followed by separation by elution with small orthophase silica gel (CH_2_Cl_2_:MeOH), and finally purified using PHPLC to give monomeric compound **17** (3.0 mg, V_methanol:water_ = 43:57, *t*_R_ = 12 min).

After purification to obtain the monomeric compounds, the organic solvent was evaporated and weighed, and a suitable deuterium substitute reagent was selected for dissolution and sent for testing, and TMS was used as an internal standard.

### 2.5. Chemical Structure Analysis

The results of bioactivity-guided isolation indicated that the major antioxidant compounds in the ethanolic extracts of *C. fascicularis* may be present in Fr. (A–G). Fr. (D–I) is a fraction of *C. fascicularis* with antimicrobial activity. The structures of compounds were determined by spectral analysis and nuclear magnetic resonance (NMR) combined with the available literature on secondary metabolites of *C. fascicularis* leaves, which afforded 17 compounds, including fours phenolics (**1**, **3**–**4**, **14**), a phenylpropane (**2**), five terpenoids (**5**–**7**, **12**, **15**), four flavonoids and flavonoid glycosides (**8**–**10**, **16**), and two lignins (**13**, **17**). Compounds **4**–**7**, **13**–**15**, and **17** were isolated from the genus *Camellia* for the first time. The remaining compounds were also isolated from this plant for the first time.

**Figure 1:**
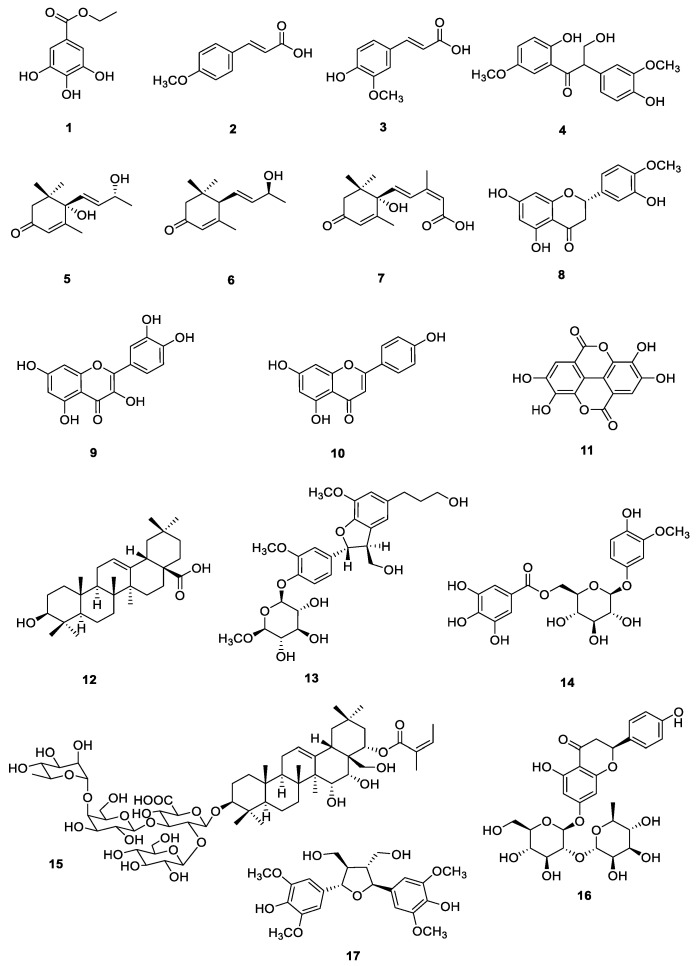
Structures of compounds **1**–**17**.

The structures in Figure 1 are of the following known compounds: ethyl gallate (**1**) [18], 4-methoxycinnamic acid (**2**) [19], ferulic acid (**3**) [20], evofolin B (**4**) [18], vomifoliol (**5**) [21], (6*R*,7*E*,9*S*)-9-hydroxy-4,7-megastigmadien-3-one (**6**) [22], (2-*cis*,4-*trans*)-abscisic acid (**7**) [23], hesperetin (**8**) [24], quercetin (**9**) [25], apigenin (**10**) [26], ellagic acid (**11**) [27], oleanolic acid (**12**) [28], dihydrodehydrodiconiferyl alcohol-4-*O*-β-D-glucopyranoside (**13**) [29], 3-Methoxy-4-hydroxyphenol 1-*O*-β-D-(6′-*O*-galloyl)-glucopyranoside (**14**) [30], 3-*O*-α-L-rhamnopyranosyl-(1→4)-β-D-galactopyranosyl-(1→3)-[β-D-glucopyranosyl-(1→2)]-β-D-glucuronopyranosyl 22-*O*-angeloyl-A1-barrigenol (**15**) [31], naringin (**16**) [32], and icario A2 (**17**) [33].

Ethyl gallate (**1**): white powder, HRESIMS *m*/*z* 199.0675 [M + H]^+^ (calcd. for C_9_H_10_O_5_); ^1^H NMR (500 MHz, methanol-*d*_4_) δ_H_ 7.06 (2H, s, H-2, 6), 4.26 (2H, d, *J* = 7.1 Hz, H-8), 1.32 (3H, t, *J* = 7.2 Hz, H-9). ^13^C NMR (126 MHz, methanol-*d*_4_) δ_C_ 168.7 (C-7), 146.6 (C-3, 5), 139.8 (C-4), 121.9 (C-7), 110.2 (C-2, 6), 61.8 (C-8), 14.7 (C-9).

4-Methoxycinnamic acid (**2**): white powder, HRESIMS *m*/*z* 179.0721 [M + H]^+^ (calcd. for C_10_H_10_O_3_); ^1^H NMR (500 MHz, methanol-*d*_4_) δ_H_ 7.62 (1H, d, *J* = 15.9 Hz, H-7), 7.53 (1H, d, *J* = 8.8 Hz, H-2, 6), 6.94 (1H, d, *J* = 8.8 Hz, H-3, 5), 6.32 (1H, d, *J* = 15.9 Hz, H-8), 3.82 (2H, s, OMe). ^13^C NMR (126 MHz, methanol-*d*_4_) δ_C_ 170.8 (C-9), 163.1 (C-4), 146.2 (C-7), 130.9 (C-2, 6), 128.4 (C-1), 116.6 (C-8), 115.4 (C-3, 5), 55.9 (4-OMe).

Ferulic acid (**3**): colourless needle crystal, HRESIMS *m*/*z* 195.0672 [M + H]^+^ (calcd. for C_10_H_10_O_4_); ^1^H NMR (500 MHz, methanol-*d*_4_) δ_H_ 7.58 (1H, d, *J* = 16.0 Hz, H-7), 7.15 (1H, d, *J* = 2.0 Hz, H-2), 7.04 (1H, dd, *J* = 8.2, 2.0 Hz, H-6), 6.80 (1H, d, *J* = 8.2 Hz, H-5), 6.29 (1H, d, *J* = 15.9 Hz, H-8), 3.87 (3H, s, 3-OMe). ^13^C NMR (126 MHz, methanol-*d*_4_) δ_C_ 171.0 (C-9), 150.5 (C-3), 149.3 (C-4), 146.9 (C-7), 127.8 (C-1), 123.9 (C-6), 116.4 (C-5), 115.9 (C-8), 111.6 (C-2), 56.4 (3-OMe).

Evofolin B (**4**): brownish yellow powder, HRESIMS *m*/*z* 319.1219 [M + H]^+^ (calcd. for C_17_H_18_O_6_); ^1^H NMR (500 MHz, methanol-*d*_4_) δ_H_ 7.62 (1H, dd, *J* = 8.4, 2.0 Hz, H-6), 7.57 (1H, d, *J* = 2.0 Hz, H-2), 6.90 (1H, d, *J* = 1.9 Hz, H-2′), 6.80 (1H, d, *J* = 8.3 Hz, H-5), 6.76 (1H, dd, *J* = 8.2, 2.0 Hz, H-6′), 6.73 (1H, d, *J* = 8.1 Hz, H-5′), 4.76 (1H, dd, *J* = 8.8, 5.2 Hz, H-7′), 4.25 (1H, dd, *J* = 10.7, 8.8 Hz, H-8′), 3.87 (3H, s, 3-OMe), 3.83 (3H, s, 3′-OMe), 3.71 (1H, dd, *J* = 10.6, 5.2 Hz, H-8′). ^13^C NMR (126 MHz, methanol-*d*_4_) δ_C_ 199.6 (C-7), 153.2 (C-3), 149.1 (C-6), 149.3 (C-4), 149.0 (C-4′), 130.5 (C-1), 129.9 (C-1′), 124.4 (C-6), 121.1 (C-6′), 116.6 (C-5′), 115.7 (C-5), 112.8 (C-2), 112.6 (C-2‘), 65.5 (C-8′), 56.4 (3-OMe), 56.3 (3′-OMe).

Vomifoliol (**5**): white crystal, HRESIMS *m*/*z* 247.1358 [M + Na]^+^ (calcd. for C_13_H_20_O_3_); ^1^H NMR (500 MHz, methanol-*d*_4_) δ_H_ 5.89–5.87 (1H, m, H-4), 5.81–5.75 (2H, m, H-7,8), 4.37–4.27 (1H, m, H-9), 2.49 (1H, d, *J* = 16.9 Hz, H-2a), 2.16 (1H d, *J* = 17.1 Hz, H-2b), 1.92 (3H, d, *J* = 1.3 Hz, H-11), 1.25 (3H, d, *J* = 6.5 Hz, H-10), 1.05 (3H, s, H-12), 1.02 (3H, s, H-13). ^13^C NMR (126 MHz, methanol-*d*_4_) δ_C_ 200.7 (C-3), 166.9 (C-5), 136.4 (C-8), 129.5 (C-7), 126.6 (C-4), 79.5 (C-6), 68.1 (C-9), 50.2 (C-2), 41.9 (C-1), 24.0 (C-13), 23.3 (C-10), 22.9 (C-12), 19.0, (C-11).

(6*R*,7*E*,9*S*)-9-Hydroxy-4,7-megastigmadien-3-one (**6**): colourless oily, HRESIMS *m*/*z* 209.1586 [M + H]^+^ (calcd. for C_13_H_20_O_2_); ^1^H NMR (500 MHz, methanol-*d*_4_) δ_H_ 5.88 (1H, s, H-4), 5.70 (1H, dd, *J* = 15.3, 5.8 Hz, H-8) 5.58 (1H, dd, *J* = 15.3, 9.2 Hz, H-7), 4.33–4.18 (1H, m, H-9), 2.67 (d, *J* = 9.2 Hz, 1H), 2.40 (1H, d, *J* = 16.8 Hz, H-2b), 2.05 (1H, d, *J* = 16.8 Hz, H-2a), 1.94 (3H, m, H-13), 1.24 (3H, d, *J* = 9.4 Hz, H-10), 1.03 (3H, s, H-11), 1.00 (3H, s, H-12). ^13^C NMR (126 MHz, methanol-*d*_4_) δ_C_ 202.2 (C-3), 166.2 (C-5), 140.4 (C-8), 127.4 (C-7), 126.3 (C-4), 68.9 (C-9), 56.8 (C-6), 37.3 (C-1), 27.5 (C-11), 23.9 (C-13), 23.9 (C-10).

(2-*cis*,4-*trans*)-Abscisic acid (**7**): white amorphous powder, HRESIMS *m*/*z* 287.1307 [M + H]^+^ (calcd. for C_15_H_20_O_4_); ^1^H NMR (500 MHz, methanol-*d*_4_) δ_H_ 7.76 (1H, d, *J* = 16.2 Hz, H-4), 6.22 (1H, d, *J* = 16.2 Hz, H-5), 5.92 (1H, s, H-8), 5.75 (1H, s, H-2), 2.53 (1H, d, *J* = 16.9 Hz, H-10a), 2.18 (1H, d, *J* = 16.8 Hz, H-10b), 2.02 (3H, d, *J* = 1.2 Hz, H-15), 1.93 (3H, d, *J* = 1.4 Hz, H-14), 1.06 (3H, s, H-12), 1.02 (3H, s, H-13). ^13^C NMR (126 MHz, methanol-*d*_4_) δ_C_ 201.4 (C-9), 166.9 (C-1, 3), 150.4 (C-7), 137.8 (C-5), 129.8 (C-4), 127.8 (C-8), 120.8 (C-2), 80.9 (C-6), 51.0 (C-10), 43.2 (C-11), 25.0 (C-12), 23.9 (C-13), 21.5 (C-15), 20.0 (C-14).

Hesperetin (**8**): pale yellow crystals, HRESIMS *m*/*z* 303.1002 [M + H]^+^ (calcd. for C_16_H_14_O_6_); ^1^H NMR (500 MHz, methanol-*d*_4_) δ_H_ 6.96 (1H, d, *J* = 2.3 Hz, H-2′), 6.94 (s, 1H), 6.91 (1H, dd, *J* = 8.3, 2.1 Hz, H-5′), 5.91 (1H, d, *J* = 2.2 Hz, H-6), 5.89 (1H, d, *J* = 2.2 Hz, H-8), 5.33 (1H, dd, *J* = 12.7, 3.1 Hz, H-8), 3.87 (3H, s, OMe), 3.07 (1H, dd, *J* = 17.1, 12.7 Hz, H-3b), 2.72 (1H, dd, *J* = 17.1, 3.1 Hz, H-3a). ^13^C NMR (126 MHz, methanol-*d*_4_) δ_C_ 197.6 (C-4), 168.4 (C-7), 165.5 (C-5), 164.8 (C-9), 149.4 (C-4′), 147.8 (C-3′), 133.2 (C-1′), 119.0 (C-6′), 114.5 (C-2′), 112.6 (C-5′), 103.4 (C-10), 97.1 (C-6), 96.2 (C-8), 80.3 (C-2), 56.4 (OMe), 44.1 (C-3).

Quercetin (**9**): yellow powder, HRESIMS *m*/*z* 301.0496 [M − H]^−^ (calcd. for C_15_H_10_O_7_); ^1^H NMR (500 MHz, methanol-*d*_4_) δ_H_ 7.71 (1H, d, *J* = 2.2 Hz, H-2′), 7.60 (1H, dd, *J* = 8.5, 2.2 Hz, H-6′), 6.86 (1H, d, *J* = 8.5 Hz, H-5′), 6.36 (1H, d, *J* = 2.1 Hz, H-8), 6.15 (1H, d, *J* = 2.1 Hz, H-6). ^13^C NMR (126 MHz, methanol-*d*_4_) δ_C_ 177.3 (C-4), 165.6 (C-7), 162.5 (C-5), 158.2 (C-9), 148.8 (C-2), 147.9 (C-4′), 146.2 (C-3′), 137.3 (C-3), 124.2 (C-1′), 121.7 (C-6′), 116.2 (C-5′), 115.9 (C-2′), 104.5 (C-10), 99.2 (C-6), 94.4 (C-8).

Apigenin (**10**): pale yellow powder, HRESIMS *m*/*z* 271.1464 [M + H]^+^ (calcd. for C_15_H_10_O_5_); ^1^H NMR (500 MHz, DMSO-*d*_6_) δ_H_ 12.97 (1H, s, 5-OH), 7.93 (2H, d, *J* = 8.9 Hz, H-2′, 6′), 6.93 (2H, d, *J* = 8.8 Hz, H-3′, 5′), 6.78 (1H, s, H-3), 6.48 (1H, d, *J* = 2.1 Hz, H-8), 6.19 (1H, d, *J* = 2.0 Hz, H-6). ^13^C NMR (126 MHz, DMSO-*d*_6_) δ_C_ 181.9 (C-4), 164.3 (C-2), 163.9 (C-7), 161.6 (C-4′), 157.5 (C-5), 128.7 (C-2′, 6′), 121.3 (C-1′), 116.1 (C-3′, 5′), 103.9 (C-10), 99.0 (C-6), 94.1 (C-8).

Ellagic acid (**11**): pale yellow powder, HRESIMS *m*/*z* 303.1231 [M + H]^+^ (calcd. for C_14_H_6_O_8_); ^1^H NMR (500 MHz, DMSO-*d*_6_) *δ*_H_ 7.46 (2H, S, H-5). ^13^C NMR (126 MHz, DMSO-*d*_6_) *δ*_C_ 159.3 (C-7, 3′), 148.3 (C-4, 4′), 133.8 (C-2, 2′), 136.6 (C-3, 3′), 112.5 (C-6, 6′), 110.4 (C-5, 5′), 107.8 (C-1, 1′).

Oleanolic acid (**12**): white powder, HRESIMS *m*/*z* 455.3527 [M – H]^−^ (calcd. for C_30_H_48_O_3_); ^1^H NMR (500 MHz, methanol-*d*_4_) δ_H_ 5.22 (1H, t, *J* = 3.7 Hz, H-12), 3.13 (1H, dd, *J* = 11.4, 4.8 Hz, H-3a), 2.83 (1H, dd, *J* = 13.9, 4.6 Hz, H-18b), 1.14 (1H, s, 27-Me), 0.95 (3H, s, 23-Me), 0.92 (3H, s, 29-Me), 0.89 (3H, s, 24-Me), 0.80 (3H, s, 25-Me), 0.76 (3H, s, 26-Me). ^13^C NMR (126 MHz, methanol-*d*_4_) δ_C_ 182.0 (C-28), 145.4 (C-13), 123.8 (C-12), 79.9 (C-3), 56.9 (C-5), 47.8 (C-9), 47.5 (C-19), 47.4 (C-17), 43.0 (C-14), 42.9 (C-18), 40.7 (C-8), 40.0 (C-4), 38.3 (C-1), 35.0 (C-10), 34.2 (C-21), 34.0 (C-30), 33.7 (C-22), 31.8 (C-7), 29.0 (C-20), 28.9 (C-23), 28.0 (C-15), 26.6 (C-2), 24.7 (C-29), 24.2 (C-16), 24.1 (C-11), 19.7 (C-6), 17.9 (C-26), 16.5 (C-25), 16.0 (C-24).

Dihydrodehydrodiconiferyl alcohol-4-*O*-β-D-glucopyranoside (**13**): white powder, HRESIMS *m*/*z* 545.2504 [M + Na]^+^ (calcd. for C_26_H_34_O_11_); ^1^H NMR (500 MHz, methanol-*d*_4_) *δ*_H_ 7.16 (1H, d, *J* = 8.4 Hz, H-5), 7.05 (1H, d, *J* = 2.0 Hz, H-2), 6.95 (1H, dd, *J* = 8.3, 2.0 Hz, H-6), 6.75 (1H, s, H-6′), 6.73 (1H, s, 2′), 5.57 (1H, d, *J* = 5.8 Hz, H-7), 4.91 (1H, s, H, Glc-1), 3.88 (3H, s, 3′-OMe), 3.85 (3H, s, 3-OMe), 3.80 (1H, s, H-9a), 3.57 (1H, t, *J* = 6.4 Hz, H-8), 2.64 (2H, dd, *J* = 8.7, 6.7 Hz, H-7′), 1.86–1.80 (2H, m, H-8′). ^13^C NMR (126 MHz, methanol-*d*_4_) *δ*_C_ 151.1 (C-3), 147.8 (C-4), 147.7 (C-4′), 145.4 (C-3′), 138.5 (C-1), 137.3 (C-1′), 129.8 (C-5′), 119.5 (C-6), 118.2 (C-5), 118.1 (C-2′), 114.3 (C-6′), 111.3 (C-2), 102.9 (Glc, C-1), 88.6 (C-7), 78.4 (Glc, C-3), 78.0 (Glc, C-5), 75.1 (Glc, C-2), 71.5 (Glc, C-4), 65.2 (C-9), 62.7 (Glc, C-6), 62.4 (C-9′), 56.9 (3-OMe), 56.9 (3′-OMe), 55.8 (C-8), 35.9 (C-8′), 33.1 (C-7′).

3-Methoxy-4-hydroxyphenol 1-*O*-β-D-(6′-*O*-galloyl)-glucopyranoside (**14**): white powder, HRESIMS *m*/*z* 477.1048 [M + Na + H]^+^ (calcd. for C_20_H_21_O_12_); ^1^H NMR (500 MHz, methanol-*d*_4_) δ_H_ 7.10 (2H, s, H-2″, 6″), 6.70 (1H, d, *J* = 2.7 Hz, H-5), 6.62 (1H, d, *J* = 8.6 Hz, H-2), 6.57 (1H, dd, *J* = 8.6, 2.7 Hz, H-6), 4.73 (1H, d, *J* = 7.4 Hz, H-1′), 4.59 (1H, dd, *J* = 11.9, 2.1 Hz, H-6a′), 4.43 (1H, dd, *J* = 11.9, 6.7 Hz, H-6b′), 3.92–3.76 (1H, m, H-5′), 3.71 (3H, s, -OMe), 3.50–3.39 (3H, m, H-2′, 3′, 4′). ^13^C NMR (126 MHz, methanol-*d*_4_) δ_C_ 168.3 (C-7″), 152.7 (C-1), 149.2 (C-3), 146.6 (C-3″, 5″), 143.1 (C-4), 139.9 (C-4′), 121.4 (C-1′), 116.1 (C-5), 110.2 (C-2′, 6′), 110.2 (C-6), 103.9 (C-1′), 103.9 (C-2), 77.9 (C-3′), 75.7 (C-5′), 74.9 (C-2′), 71.8 (C-4′), 64.9 (C-6′), 56.3 (-OMe).

3-*O*-α-L-rhamnopyranosyl-(1→4)-β-D-galactopyranosyl-(1→3)-[β-D-glucopyranosyl-(1→2)]-β-D-glucuronopyranosyl 22-*O*-angeloyl-A1-barrigenol (**15**): white powder, HRESIMS *m*/*z* 1219.5961 [M + H]^+^ (calcd. for C_59_H_94_O_26_); ^1^H NMR (500 MHz, methanol-*d*_4_) *δ*_H_ 1.11 (1H, m, H-1a), 1.68 (1H, m, H-2), 1.65 (1H, d, *J* = 13.2 Hz, H-1b), 3.29 (1H, dd, *J* = 13.2, 4.2 Hz, H-3), 0.72 (1H, d, *J* = 12.3 Hz, H-5), 1.50–1.42 (2H, m, H-6), 1.73–1.64 (2H, m, H-7), 1.59–1.52 (1H, m, H-9), 1.97–1.84 (2H, m, H-11), 5.43 (1H, t, *J* = 3.9 Hz, H-12), 3.79–3.63 (1H, m, H-15), 3.87 (1H, d, *J* = 4.9 Hz, H-17), 2.49 (1H, dd, *J* = 13.2, 4.6 Hz, H-18), 2.41 (1H, t, *J* = 13.3 Hz, H-19), 1.09–0.92 (1H, m, H-19), 1.51 (1H, d, *J* = 12.3 Hz, H-21), 2.31 (1H, t, *J* = 12.4 Hz, H-21), 5.45 (1H, dd, *J* = 12.0, 5.0 Hz, H-18), 1.05 (3H, s, H-23), 0.85 (3H, s, H-24), 0.95 (3H, s, H-29), 1.07 (3H, s, H-26), 1.36 (3H, s, H-27), 3.11 (2H, d, *J* = 11.3 Hz, H-21), 0.89 (3H, s, H-27), 1.03 (3H, s, H-30), 6.04 (1H, s, H-3′), 1.95 (3H, d, *J* = 7.1 Hz, H-4′), 1.88 (3H, d, *J* = 1.9 Hz, H-5′), 4.43 (1H, d, *J* = 8.6 Hz, GlcA, H-1), 3.93 (1H, d, *J* = 8.4 Hz, GlcA, H-2), 4.10 (1H, d, *J* = 8.8 Hz, GlcA, H-3), 3.65 (1H, d, *J* = 9.6 Hz, GlcA, H-4), 3.79 (1H, d, *J* = 9.4 Hz, GlcA, H-5), 4.89 (1H, d, *J* = 6.8 Hz, Glc, H-1), 3.23 (1H, d, *J* = 8.8 Hz, Glc, H-2), 3.33 (1H, d, *J* = 8.9 Hz, GlcA, H-3), 3.10 (1H, t, *J* = 8.8 Hz, Glc, H-4), 3.40–3.33 (1H, m, Glc, H-5), 3.58 (1H, dd, *J* = 12.4, 4.8Hz, Glc, H-6), 5.11 (1H, d, *J* = 8.1 Hz, Gal, H-1), 3.79 (1H, d, *J* = 8.8 Hz, Gal, H-2), 3.67 (1H, dd, *J* = 9.4, 3.4Hz, Gal, H-3), 3.73 (1H, d, *J* = 3.8 Hz, Gal, H-4), 3.48 (1H, dd, *J* = 8.2, 3.7Hz, Gal, H-5), 3.63 (1H, dd, *J* = 11.4, 3.8Hz, Gal, H-6a), 3.80 (1H, dd, *J* = 11.7, 7.8Hz, Gal, H-6b), 5.23 (1H, s, Rha, H-1), 3.91 (1H, d, *J* = 1.8 Hz, Rha, H-2), 3.72 (1H, dd, *J* = 9.4, 3.1 Hz, Rha, H-3), 3.44 (1H, t, *J* = 8.3 Hz, Rha, H-4), 4.08 (1H, dd, *J* = 9.2, 5.7Hz, Rha, H-5), 1.25 (1H, d, *J* = 6.4 Hz, Rha, H-6), ^13^C NMR (126 MHz, methanol-*d*_4_) δ_C_ 170.8 (GlcA C-6), 169.4 (C-1′), 144.3 (C-13), 129.8 (C-2′), 126.1 (C-12), 105.9 (GlcA C-1), 103.0 (Glc C-1), 102.7 (Rha C-1), 101.5 (Gal C-1), 92.1 (C-3), 82.7 (GlcA C-6), 79.7 (GlcA C-2), 78.8 (Glc C-5), 78.5 (Gal C-5), 77.7 (Glc C-3), 76.8 (Gal C-2), 76.7 (Glc C-2), 76.0 (GlcA C-5), 75.4 (C-16), 75.2 (Gal C-4), 74.6 (Rha C-4), 73.2 (C-22), 73.2 (Rha C-2), 72.7 (Glc C-4), 72.1 (Gal C-3), 71.4 (GlcA C-4), 71.4 (Rha C-3), 70.8 (Rha C-5), 68.4 (C-15), 63.7 (C-28), 63.7 (Glc C-6), 62.7 (Gal C-6), 56.5 (C-5), 49.5 (C-14), 49.0 (C-19), 47.8 (C-9), 47.6 (C-19), 45.5 (C-17), 42.3 (C-18), 42.1 (C-8), 41.7 (C-21), 40.2 (C-1), 40.0 (C-1), 37.7 (C-10), 37.0 (C-7), 33.4 (C-29), 32.3 (C-20), 28.2 (C-23), 26.9 (C-2), 25.6 (C-27), 25.12 (C-30), 24.6 (C-11), 20.7 (C-27), 19.4 (C-6), 17.7 (Rha C-6), 17.6 (C-26), 16.1 (C-24), 15.7 (C-25).

Naringin (**16**): yellow powder, HRESIMS *m*/*z* 579.1883 [M − H]^−^ (calcd. for C_27_H_32_O_14_); ^1^H NMR (500 MHz, methanol-*d*_4_) δ_H_ 7.29 (2H, d, *J* = 8.6 Hz, H-2′, 6′), 6.80 (2H, d, *J* = 8.6 Hz, H-3′, 5′), 6.15 (1H, d, *J* = 2.3 Hz, H-8), 6.13 (1H, d, *J* = 2.2 Hz, H-6), 5.34 (1H, dd, *J* = 13.0, 2.6 Hz, H-2), 5.23 (1H, dd, *J* = 5.1, 1.7 Hz, Rha, H-1), 5.07 (1H, dd, *J* = 9.1, 7.5 Hz, Glc, H-1), 3.94–3.32 (10H, m, Sugar-H), 3.19–3.08 (1H, m, H-3), 2.77–2.67 (1H, m, H-3), 1.27 (3H, d, *J* = 6.2 Hz, Me). ^13^C NMR (126 MHz, methanol-*d*_4_) δ_C_ 198.8 (C-4), 166.7 (C-7), 165.1 (C-5), 164.8 (C-5), 164.8 (C-9), 159.3 (C-4′), 130.9 (C-1′), 129.4 (C-2′, 6′), 116.5 (C-3′, 5′), 105.1 (C-10), 102.7 (Glc, C-1), 99.5 (Rha, C-1), 98.0 (C-6), 96.9 (C-8), 80.8 (C-2), 79.3 (Glc, C-2), 79.1 (Glc, C-3), 78.3 (Glc, C-5), 74.1 (Rha, C-4), 72.3 (Rha, C-2), 72.3 (Rha, C-3), 71.4 (Glc, C-4), 70.2 (Rha, C-5), 62.4 (Glc, C-6), 44.0 (C-3), 18.4 (Rha, C-6).

Icario A2 (**17**): pale yellow powder, HRESIMS *m*/*z* 435.1645 [M − H]^−^ (calcd. for C_22_H_28_O_9_); ^1^H NMR (500 MHz, methanol-*d*_4_) δ_H_ 6.76 (4H, s, H-2, 2′, 6, 6′), 4.97 (1H, d, *J* = 8.5 Hz, H-7, 7′), 3.88 (12H, s, H-3, 3′, 5, 5′-OMe), 3.73–3.65 (4H, m, H-9, 9′), 2.36–2.28 (2H, m, H-8, 8′). ^13^C NMR (126 MHz, methanol-*d*_4_) δ_C_ 149.3 (C-3, 3′, 5, 5′), 136.2 (C-4, 4′), 134.3 (C-1, 1′), 104.9 (C-2, 2′, 6, 6′), 84.6 (C-7, 7′), 61.69 (C-9, 9′), 56.8 (-OMe), 55.2 (C-8, 8′).

### 2.6. Antioxidant Activity

#### 2.6.1. DPPH Assay

For determining the DPPH free radical-scavenging activities of the EtOAc extract of *C. fascicularis*, different fractions were assessed according to the method described by Jiang et al. [34]. The concentration gradient of the EtOAc extract and the different fractions was 0.01–10.0 mg/mL, and the concentration gradient of V_C_ was 5–30 µg/mL. Initially, the samples were dissolved in DMSO to different concentrations, and the DPPH was dissolved in PBS (0.1 M, pH 6.8) to 0.1 mM. Then, 300 µL DPPH solution and 300 µL sample were mixed in each well of a 2 mL Eppendorf (EP) tube well plate and incubated for 30 min at room temperature in the dark. The absorbance was measured using a microplate reader at 517 nm, and ascorbic acid was used as a positive control. All tests were performed in triplicate, and the obtained results were processed by analysis of variance (ANOVA) with 95% confidence (*p* ≤ 0.05). Results are expressed as half-maximal inhibitory concentration (IC_50_).

The percentage radical scavenging rate (%) of each test sample for DPPH was calculated as follows (1):

Radical scavenging rate (%) = [A_blank_ − (A_sample_ − A_control_)]/A_blank_ × 100% (1)

#### 2.6.2. ABTS Assay

The ABTS free radical-scavenging capacity of all isolated compounds was measured using a previous method with minor modifications [35]. The experiment was conducted using a 96-well plate, and each well had a total volume of 210 µL. Equal volumes of ABTS solution (7 mM) and potassium persulfate solution (5 mM) were mixed evenly and left to react at room temperature for 12 h in the dark to obtain the ABTS radical cation. The mixture was then diluted with anhydrous methanol to achieve an absorbance value of about 0.7 ± 0.02 units at 734 nm. Then, 180 µL of ABTS working solution was added to each well, followed by 30 µL samples of varying concentrations (0.01–10.0 mg/mL) that were dissolved and diluted with DMSO. After mixing well, the samples were incubated at room temperature for 6 min away from light. After that, the absorbance values of each well were measured at 734 nm using a microplate reader, and the results were obtained from a minimum of three independent experiments. Ascorbic acid was used as the positive control. DMSO was used to replace the sample solution as a blank, and absolute methanol was used to replace the ABTS solution as a control. All tests were performed in triplicate, and the obtained results were processed by ANOVA with 95% confidence (*p* ≤ 0.05). Results are expressed as IC_50_.

The percentage radical cation-scavenging rate (%) of each test sample for ABTS was calculated as follows (2):

Radical cation-scavenging rate (%) = [A_blank_ − (A_sample_ − A_control_)]/A_blank_ × 100% (2)

#### 2.6.3. Ferric Ion-Reducing Antioxidant Power (FRAP) Assay

The FRAP of all isolated compounds was measured using a slightly modified methodology [36]. The acetate buffer (0.3 M, pH 3.6), TPTZ (2,4,6-tris(2-pyridyl)-S-triazine) solution (10 mM) in 40 mM HCl, and ferric chloride aqueous solution (20 mM) were uniformly mixed in a volume ratio of 10:1:1 to obtain the FRAP working solution. The FRAP reserve is pre-warmed for 10 min at 37 °C in a constant temperature water bath before use. The experiment was started in an EP tube for the reaction. Then, 2 mL of FRAP working solution was added to each well, followed by 150 µL of sample solution 0.5 mg/mL. Then, the mixture was stirred well and left to react at room temperature for 30 min. Then, 200 µL of the reaction solution was added into the 96-well plate. The absorbance values of each well were measured at 593 nm using a microplate reader, and the results were obtained from at least three independent experiments. The sample solution was replaced with DMSO as a blank solution, and ascorbic acid was used as a positive control, which results in absorbance.

### 2.7. Antimicrobial Activity

The antibacterial activity capacity of all isolated compounds was measured using a previous method with minor modifications [37,38]. Antibacterial compound solution composition: dissolve the drug in DMSO such that its mass concentration was 0.5 mg/mL. Preparation of the bacterial solution for testing: remove the frozen bacteria from the −80 °C low-temperature storage box to thaw at room temperature, and Nutrient Broth (NB) medium sterilised at 37 °C in a shaking bed was used for the overnight culture. Then, 2 mL of the overnight culture was taken and inoculated into NB medium, incubated at 37 °C until A_600_ = 0.5, diluted 100 times with NB medium, and set aside. Microdilution method: a sterile 96-well plate was taken, 75 μL of NB medium dilution solution was added to A2–A11 wells, and 75 μL of compound solution was added to A1–A2 wells. After gradient dilution from A2, an 8-channel pipette was used to pipette 75 μL of each compound dilution into a 96-well plate so that the final concentration of compounds in the 96-well plate was in the range of 500 to 1.95 µg/mL. The inoculated 96-well plates were incubated at 37 °C for 12 h. After inoculation, the 96-well plates were incubated at 37 °C for 16 h to observe the growth, and the minimum inhibitory concentration (MIC) was calculated by measuring A_600_ with an enzyme marker. All tests were performed in triplicate, and the obtained results were processed by ANOVA with 95% confidence (*p* ≤ 0.05).

## 3. Results and Discussion

### 3.1. Antioxidant Activity of the EtOAc Extract and Fr. (A–I)

Oxidative stress (OS) refers to the imbalance between oxidation and antioxidation in the body. Studies have found that oxidative stress is related to a variety of diseases, such as cardiovascular diseases, diabetes, and metabolic disorders [39]. Therefore, the EtOAc extract and Fr. (A–I) of *C. fascicularis* were evaluated for their antioxidant activities using DPPH, ABTS, and FRAP assays to obtain their IC_50_ concentrations. As shown in Table 2, the EtOAc extract had antioxidant activity. This result supported the benefits of using *C. fascicularis* in herbs and food. Furthermore, in the DPPH and ABTS tests, we found that the EtOAc extract and the different fractions were capable of scavenging DPPH and ABTS free radicals in a concentration-dependent manner. The results showed that the DPPH free radical-scavenging ability was D > F> A > G > E > B* > I > H, the ABTS free radical-scavenging ability was D > B* > E > F > A > I > G > H, the FRAP free radical-scavenging ability was D > B* > E > A > F > G > I > H, and the scavenging capacity of DPPH and ABTS was related to the accumulative effect of fractions. DPPH, ABTS, and FRAP assays showed that the antioxidant activity of Fr. (A–F) had obvious antioxidant activity, Fr. (G) had moderate antioxidant activity, and Fr. (H–I) had no significant antioxidant activity. The results suggested that the main antioxidant compounds of the EtOAc extract of *C. fascicularis* might be present in the Fr. (A–G).

DPPH, ABTS, and FRAP assays also showed some differences in the experimental results, which may be attributed to the different mechanisms of scavenging free radicals. The main pathway of scavenging free radicals is through the transfer of electrons from the antioxidant or the transfer of hydrogen to the free radicals [40]. ABTS can scavenge through the transfer of both electrons and hydrogen, whereas the scavenging of free radicals by DPPH is mainly through the transfer of hydrogen to the free radicals through the antioxidant, and this effect dominates only in strong hydrogen-bonding solvents such as methanol. DPPH scavenges free radicals mainly by transferring hydrogen from the antioxidant to the free radicals, and electron transfer is dominant only in strongly hydrogen-bonded solvents such as methanol [41].

### 3.2. Antimicrobial Activities of the EtOAc Extract and Fr. (A–I)

The application of plant extracts as natural antimicrobial agents in the food industry is a growing trend [42]. The antimicrobial activity testing of the samples was performed against microorganism strains from the laboratory collection. The Gram-positive bacteria used for the tests were *Staphylococcus aureus* (ATCC 6538). The Gram-negative bacteria were *Escherichia coli* (ATCC 6538) and *Pseudomonas aeruginosa* (CGMCC 1.10712). The antimicrobial activity of *C. fascicularis* has not been reported thus far, but the antimicrobial activity of *Camellia* has been proven, and *Camellia* has good antimicrobial activity [43,44,45]. The findings provide additional evidence to support the high antibacterial activity of secondary metabolites derived from *C. fascicularis*. Therefore, the EtOAc extract and Fr. (A–I) of *C. fascicularis* were evaluated for their antimicrobial activity according to microbroth dilution method and MIC. The concentration of the EtOAc extract and the different fractions was 20 mg/mL.

As shown in Table 3, the MICs of the EtOAc extract against *E. coli*, *S. aureus,* and *P. aeruginosa* were all greater than 20 mg/mL. Fr. (E–F, I) showed good inhibition of *E. coli* (MIC 20 mg/mL), Fr. (E, G–I) showed good inhibition of *S. aureus* (MIC 20 mg/mL), and Fr. (D–E, H–I) showed good inhibition of *P. aeruginosa* (MIC: 10 mg/mL). However, as a whole, the EtOAc extract and the different fractions had a higher inhibitory effect on *P. aeruginosa* than on *E. coli* and *S. aureus*. Fr. (D–I) is the fraction of the EtOAc extract of *C. fascicularis* with antibacterial activity. It is more interesting to note that Fr. (D–G) has not only good antioxidant activity, but also its antimicrobial activity is also prominent, which may be related to the active components such as phenolic acids, flavonoids, lignans, terpenoids, etc. [46,47,48]. However, Fr. (H–I) has poor antioxidant activity and prominent antimicrobial activity, suggesting that there may be secondary metabolites in this fraction that are worthy of further exploration.

### 3.3. Antioxidant Activity of the Compounds

In the ABTS assay, the antioxidant activity is measured as the ability of test compounds to decrease the colour by reacting directly with the radical ABTS [49]. Methods such as the ABTS assay were used to evaluate antioxidant activities of all the isolates in vitro, and the results are shown in Table 4. The results of these experiments showed that compounds **1**, **3**, **9**, **11,** and **17** exhibited higher antioxidant activity than the positive control drug, ascorbic acid, and the antioxidant activity of compounds **4**, **8**, **10,** and **13** was slightly lower than that of ascorbic acid. The other compounds had weaker or no significant antioxidant activities. This is also the first report of antioxidant activity of compounds **4**–**7**, **13**–**15**, and **17**. This finding once again confirms that flavonoids [50,51,52], lignans [53,54,55], and phenolic acid [56] compounds have good antioxidant activities.

The compounds **1**, **3**–**4**, and **14** are phenolics; however, compounds **1**, **3**–**4**, and **14** are phenolic acids with hydroxybenzoic acid as the parent compound, and compound **4** has significantly lower antioxidant activity than compounds **1**–**3** and **14,** and the introduction of *O*-hydroxy and *O*-methoxy groups promotes the antioxidant activity of phenolic acids [57]. Compound **2** has no active site in its structure, which contributes to its poor antioxidant activity. Compounds **5**–**7**, **12,** and **15** are terpenoids, and compounds **5**–**7** are β-ionone derivatives, which have certain antioxidant activity and may be the unique aromatic component of *C. fascicularis*. Meanwhile, the development of β-ionone derivatives of curcuminoids can be prioritised by the substitution of meso and neighbouring substituent derivatives and the replacement of benzene’s second substituent group, so as to obtain a better biological activity. Compounds **12** and **17** are triterpenoids, but the antioxidant activity of compound **17** was significantly lower than that of compound **12**, probably due to the introduction of the sugar group and the substitution of the active site to reduce the activity of the compounds.

Compound **9** exhibits significantly higher antioxidant activity compared to compounds **8** and **10**. An analysis of the structure–activity relationship for these three flavonoids shows that the free radical-scavenging activity is directly correlated with the number of hydroxyl groups on the B ring. An increase in the number of hydroxyl groups on the B ring leads to enhanced free radical-scavenging activity. Additionally, it is worth noting that C-3 possesses an alcohol hydroxyl group that demonstrates greater stability and reduced susceptibility to electron loss when compared to a phenolic hydroxyl group. This characteristic contributes to the increased water solubility of compounds without significantly impacting their antioxidant activity. These findings are consistent with previous analyses of the literature on the relationship between the antioxidant activities and conformations of flavonoids [58,59,60]. Meanwhile, there were differences in the antioxidant activities of compounds **8**, **9,** and **16**, which were mainly related to the number and position of hydroxyl groups and the spatial site resistance of the glycosides [61]. The large difference in the antioxidant activity of compounds **13** and **17** may be due to the presence of methoxy in the neighbourhood of the phenolic hydroxyl group to provide power, which positively affects the antioxidant activity of lignans [62]. Compound **13** may be the main component of *C. fascicularis*, giving rise to its bitter flavour [63]. This suggests that flavonoids, phenols, lignans, and terpenoids are the major antioxidant secondary metabolites in *C. fascicularis*.

### 3.4. Antibacterial Activity of the Compounds

Most of the compounds with antimicrobial activity in plants are plant secondary metabolites, and the most abundant species can be divided into polysaccharides, phenols, alkaloids, terpenoids, etc., and their rich variety and diverse chemical structures can produce antimicrobial activity through a variety of pathways, and the main antimicrobial mechanisms include the disruption of the cellular structure, regulation of gene expression, inhibition of bacterial metabolic activity, and alteration in the cell membrane potential [64,65].

As shown in Table 5, the results of the antimicrobial activity test showed that compounds **1**–**17** inhibited *E. coli* to a certain extent at a concentration of 500 µg/mL, among which the antimicrobial activity of compound **11** was lower than that of other compounds. Compounds **1**–**3** and **5**–**11** showed some degree of inhibition against *S. aureus* at 250–500 µg/mL, and among them, compound **12** showed better antibacterial activity than the other compounds, but this activity was still weaker compared to that of the positive control drug. The antibacterial activity of compounds **4**, **7**, and **11**–**12** against *P. aeruginosa* was comparable to that of the positive control drug tetracycline (MIC: 125.00 µg/mL) and superior to that of penicillin (MIC: 250.00 ug/mL), whereas the antibacterial activity of compounds **1**, **3**, **5**–**6**, **8**–**10**, and **14**–**17** against *P. aeruginosa* was comparable to that of the positive control drug penicillin (MIC: 250.00 µg/mL) and weaker than that of penicillin (MIC: 250.00 µg/mL) and tetracycline (MIC: 125.00 µg/mL). Polyphenols play an important role in plants and are an important material basis for protecting plants from foreign invasion and injury such as those caused by parasites and pathogenic bacteria [66]. It has been shown that the increase in rhamnose content in the analysed plants improves the antimicrobial properties of the polysaccharides, which may also be the reason for the antimicrobial activity of compound **15** to some degree [67]. Usually ring-forming monoterpenes are more active than acyclic ones, and different substituents produce different effects on the antimicrobial activity of terpenoids [68]. This suggests that flavonoids, phenolics, and terpenoids are the main secondary metabolites producing the antimicrobial activity of *C. fascicularis*. The antimicrobial effects of compounds **1**–**17** were essentially consistent with the those observed in the antimicrobial-directed activity screening, and they inhibited *P. aeruginosa* better compared to *E. coli* and *S. aureus*.

## 4. Conclusions

The secondary metabolites of the leaves of *C. fascicularis* were investigated. The results of bioactivity-guided isolation indicated that the major antioxidant compounds in the ethanolic extracts of *C. fascicularis* may be present in *C. fascicularis* Fr. (A–G). Fr. (D–I) is a fraction of *C. fascicularis* with antimicrobial activity, containing 17 compounds, including fours phenolics (**1**,**3**–**4**, **14**), a phenylpropane (**2**), five terpenoids (**5**–**7**, **12**, **15**), four flavonoids and flavonoid glycosides (**8**–**10**, **16**), and two lignins (**13**, **17**). Compounds **4**–**7**, **13**–**15**, and **17** were isolated from the genus *Camellia* for the first time. The remaining compounds were also isolated from *C. fascicularis* for the first time. This is also the first report of the antioxidant and antimicrobial activities of compounds **5**–**7**, **13**–**15**, and **17**. The antioxidant and antimicrobial activities of the isolated and obtained compounds were determined. These activities suggest that flavonoids, phenols, lignans, and terpenoids are the main secondary metabolites that produce the antioxidant and antibacterial activities of *C. fascicularis*. The results of the study enriched the variety of secondary metabolites of *C. fascicularis*, laid the foundation for further research on the pharmacological efficacy and biological activity of this plant, and provided reference and theoretical basis for the development and utilisation of this plant resource.

## Figures and Tables

**Table 1:** Weight in (Fr. A–I) of *C. fasciculata*.

Fractions (g)								
A	^1^ B*	D	E	F	G	H	I	Fr. II–III
6.16	2.61	4.52	7.16	5.63	9.53	2.81	5.73	44.15/45.00

“^1^ B*” was obtained by combining the B and C fractions.

**Table 2:** Antioxidant activities of EtOAc extract and fractions of *C. fascicularis*.

Component	Antioxidant Activities		
	DPPH IC_50_/(µg/mL)	ABTS IC_50_/(µg/mL)	FRAP (500 µg/mL)
A	50.33 ± 3.80 ^fg^	253.13 ± 8.40 ^d^	0.60 ± 0.04 ^e^
^1^ B*	97.43 ± 4.67 ^c^	41.04 ± 3.73 ^ef^	1.00 ± 0.01 ^c^
D	22.11 ± 1.30 ^h^	32.31 ± 3.30 ^ef^	1.15 ± 0.04 ^b^
E	89.45 ± 4.46 ^cd^	67.86 ± 2.12 ^ef^	0.67 ± 0.02 ^d^
F	34.60 ± 2.62 ^gh^	134.05 ± 10.49 ^e^	0.39 ± 0.01 ^f^
G	74.70 ± 3.43 ^de^	690.70 ± 26.41 ^b^	0.19 ± 0.01 ^g^
H	836.90 ± 32.06 ^a^	2237.67 ± 231.83 ^a^	0.11 ± 0.00 ^h^
I	350.73 ± 15.99 ^b^	406.60 ± 3.30 ^c^	0.17 ± 0.02 ^gh^
EtOAc	65.79 ± 4.41 ^ef^	330.61 ± 16.72 ^cd^	1.00 ± 0.07 ^c^
^2^ Tetracycline	28.91 ± 0.68 ^h^	13.51 ± 0.40 ^f^	2.17 ± 0.12 ^a^

“^1^ B*” was obtained by combining the B and C fractions. “^2^”, positive control. Values accompanied by different letters are significantly different (*p* ≤ 0.05).

**Table 3:** Antibacterial activity of EtOAc extract and fractions in *C. fascicularis*.

Component	MIC mg/mL		
	*E. coli*	*S. aureus*	*P. aeruginosa*
A	>20.00	>20.00	>20.00
B*	>20.00	>20.00	>20.00
D	>20.00	>20.00	10.00
E	20.00	20.00	10.00
F	20.00	>20.00	10.00
G	>20.00	20.00	>20.00
H	>20.00	20.00	10.00
I	20.00	20.00	10.00
EtOAc	>20.00	>20.00	>20.00
^1^ Penicillin	0.0625	0.0625	0.25
^1^ Tetracycline	0.01563	0.03125	0.125

“1”, positive control.

**Table 4:** Antioxidant activities of chemical constituents **1**–**17** in *C. fascicularis*.

Compounds	ABTS^+ B^ Assay (%)			
	500 µg/mL	100 µg/mL	50 µg/mL	10 µg/mL
1	98.88 ± 0.37 ^a^	-	99.29 ± 0.83 ^a^	24.77 ± 2.14 ^d^
2	6.84 ± 2.12 ^j^	-	-	-
3	99.74 ± 0.68 ^a^	-	100.79 ± 1.66 ^a^	53.96 ± 1.89 ^a^
4	89.18 ± 2.58 ^c^	58.48 ± 0.95 ^c^	-	-
5	54.80 ± 2.58 ^i^	-		
6	57.43 ± 1.81 ^i^	-	-	-
7	55.88 ± 2.07 ^h^	-	-	-
8	86.48 ± 0.77 ^d^	66.00 ± 2.70 ^b^	-	-
9	99.47 ± 0.63 ^a^	-	100.55 ± 0.06 ^a^	47.42 ± 1.89 ^b^
10	67.18 ± 1.62 ^f^	24.72 ± 2.23 ^e^		-
11	101.03 ± 0.41 ^a^	-	100.00 ± 0.00 ^a^	39.88 ± 3.15 ^c^
12	94.12 ± 0.99 ^b^	-	-	-
13	83.34 ± 0.62 ^e^	53.38 ± 1.52 ^d^	-	-
14	99.02 ± 0.59 ^a^	-	101.11 ± 0.24 ^a^	25.71 ± 1.07 ^d^
15	4.09 ± 1.65 ^k^	-		
16	58.89 ± 0.69 ^g^	-		-
17	99.94 ± 0.51 ^a^	-	83.75 ± 0.65 ^b^	40.61 ± 1.37 ^c^
^A^ ascorbic acid	-	99.85 ± 0.03 ^a^	61.78 ± 0.69 ^c^	25.00 ± 2.06 ^d^

“A”, positive control; “B”, inhibition ratio; “-” indicates that the experiment was not performed. Values accompanied by different letters are significantly different (*p* ≤ 0.05).

**Table 5:** Antibacterial activity of chemical components **1**–**17** in *C. fascicularis*.

Compound	MIC ^b^ µg/mL		
	*E. coli*	*S. aureus*	*P. aeruginosa*
1	500.00	500.00	250.00
2	500.00	500.00	-
3	500.00	500.00	250.00
4	500.00	-	125.00
5	500.00	500.00	250.00
6	500.00	500.00	250.00
7	500.00	500.00	125.00
8	500.00	500.00	250.00
9	500.00	500.00	250.00
10	500.00	>500.00	250.00
11	>500.00	500.00	125.00
12	500.00	250.00	125.00
13	500.00	500.00	-
14	500.00	500.00	250.00
15	500.00	500.00	250.00
16	500.00	500.00	250.00
17	500.00	500.00	250.00
^a^ Penicillin	62.50	62.50	250.00
^a^ Tetracycline	15.62	31.25	125.00

“a”, positive control; “b”, minimum inhibitory concentration. “-” indicates that the experiment was not performed.

## Data Availability

The original contributions presented in the study are included in the article, further inquiries can be directed to the corresponding author.
